# Cell-free mitochondrial DNA (cf-mtDNA) in human body fluids: molecular characteristics, release mechanisms, and clinical translation—an updated review

**DOI:** 10.3389/fmolb.2026.1774015

**Published:** 2026-02-26

**Authors:** Yu Liu, Xixiang Ma, Qingmei Guan, Shunchang Zhou

**Affiliations:** 1 Department of Gynecology, Maternal and Child Health Hospital of Hubei Province, Tongji Medical College, Huazhong University of Science and Technology, Wuhan, China; 2 Laboratory Animal Center, Huazhong University of Science and Technology, Wuhan, China; 3 Department of Hubei Maternal and Child Health Hospital, Hubei University of Medicine, Wuhan, China

**Keywords:** body fluids, cf-mtDNA, molecular features, molecular markers, release mechanism

## Abstract

Since its discovery, cell-free mitochondrial DNA (cf-mtDNA) has emerged as a promising non-invasive molecular marker for disease diagnosis and prognosis. However, the biological origins of cf-mtDNA remain incompletely understood, which limits its clinical applications. This review comprehensively summarizes the molecular characteristics, release mechanisms, and diagnostic applications of cf-mtDNA. By discussing standardization of cf-mtDNA detection methods, this review aims to provide theoretical foundations for clinical translation of this emerging biomarker.

## 1 Discovery and molecular characteristics of cf-mtDNA

The journey of cf-mtDNA from serendipitous observation in 1948 to a clinically actionable biomarker in 2025 encapsulates the iterative cycle of discovery, mechanistic interrogation, and translational validation that defines modern precision medicine. The study of cf-mtDNA can be traced back to 1948, when Mandel and Metais first detected cell-free DNA (cf-DNA) in human plasma. cf-DNA comprises nuclear DNA (nDNA), mitochondrial DNA (mtDNA), viral DNA, and messenger RNA. Since this initial observation, cf-DNA has rapidly evolved into a non-invasive molecular marker with broad applications. In 2000, Zhong et al. reported the presence of specific cf-mtDNA mutations in the serum of diabetic patients (Zhong et al., 2000), marking the beginning of cf-mtDNA as a disease biomarker. Subsequent studies have confirmed the presence of cf-mtDNA in both plasma and serum. Wang et al. developed a fragmentomic assay based on aberrant cf-mtDNA patterns for non-invasive early detection of colon cancer (Wang et al., 2025). Additional investigations have demonstrated the diagnostic value of cf-mtDNA in systemic lupus erythematosus (Halfon et al., 2025), acute kidney injury (Malik, 2023) and embryo quality assessment (Steffann et al., 2015). Thus, cf-mtDNA has exhibited substantial translational potential across multiple disciplines.

Mitochondria contain their own non-chromosomal DNA—mtDNA. This 16.5 kb, non-methylated, circular double-stranded molecule encodes 22 tRNAs, 2 rRNAs, and 13 protein subunits essential for oxidative phosphorylation. These proteins constitute complexes I–IV of the electron transport chain and ATP synthase (complex V), driving mitochondrial respiration and ATP production. Studies have shown that oxidative phosphorylation capacity differs across rodent tissues, depending on mitochondrial mass and complex activity (Benard et al., 2006). A major regulatory region—the D-loop—harbors transcription promoters. Analogous to chromatin, mtDNA is packaged into nucleoids, mtDNA–protein complexes that associate with the inner mitochondrial membrane (Falkenberg et al., 2007). Over 50 nucleoid-associated proteins bind mtDNA, among which mitochondrial transcription factor A (TFAM), a high-mobility group protein, is the principal component (Lee and Han, 2017). TFAM maintains mtDNA transcription and protects against reactive oxygen species (ROS) damage (Kang and Hamasaki, 2005; Kanki et al., 2004). RNAi-mediated TFAM knockdown reduces mtDNA content in proportion to TFAM levels (Kansaku et al., 2018), and both TFAM concentration and mtDNA-binding density are modulated to achieve precise transcriptional control (Bestwick and Shadel, 2023). Despite these protective mechanisms, mtDNA repair pathways are limited compared with nDNA; for example, mitochondria lack nucleotide excision repair. Consequently, the mitochondrial genome is vulnerable to ROS and toxic insults.

The fragment size of cf-mtDNA remains controversial in research findings. Due to the lack of histone protection, mtDNA is more susceptible to degradation (Jiang et al., 2015), potentially resulting in shorter cf-mtDNA fragments compared to cf-nDNA. Studies report that cf-nDNA fragments peak at approximately 167 bp, suggesting their association with histones and circulation as intact nucleosomes in blood (Lo et al., 2010) Notably, research has demonstrated that (Wang et al., 2011) testicular germ cell cancer patients exhibit elevated levels of fragmented cf-mtDNA (79 bp and 220 bp) in plasma compared to healthy controls. Importantly, the 79 bp mtDNA fragments showed greater diagnostic value than 220 bp fragments, indicating that shorter cf-mtDNA fragments may play a critical role in human diseases.

Second-generation sequencing enables single-nucleotide resolution measurement of plasma DNA fragments. Lo et al. (Lo et al., 2010) identified a predominant plasma cf-mtDNA peak at 140 bp, shorter than the 167 bp cf-nDNA fragments. However, their fragment size analysis was technically limited by conventional DNA library preparation methods (Wang et al., 2011), which involve multiple purification steps leading to poor recovery of short DNA fragments (<100 bp). Optimized plasma DNA isolation and library preparation protocols (Zhang R. et al., 2016) have revealed significantly shorter average cf-mtDNA lengths than previously reported (Jiang et al., 2015). Collectively, these findings highlight the biological and diagnostic significance of shorter cf-mtDNA fragments, while underscoring the critical need for optimized detection methodologies to fully capture their clinical potential.

## 2 Release mechanisms of cf-mtDNA

Extracellular mtDNA exhibits diverse forms of existence. When mtDNA escapes from damaged tissues or is actively secreted by viable cells into circulation, it may exist either as free circulating mtDNA or in vesicle-encapsulated forms (including platelet-bound forms) (Torralba et al., 2016; Lindqvist et al., 2018). Following extracellular release, mtDNA responds to various cellular signals including stress and injury. If not promptly cleared, cf-mtDNA can function as an autoantigen to induce inflammatory responses. Furthermore, elevated cf-mtDNA concentrations correlate with numerous chronic diseases and may serve as prognostic biomarkers for disease progression and survival outcomes (Lindqvist et al., 2018; Shockett et al., 2016; Pyle et al., 2015).

The high mutation rate and low recombination frequency of mtDNA have driven the evolution of unique natural selection mechanisms in female germ cells to prevent accumulation of deleterious mutations. Toby Lieber et al. (Li et al., 2019) demonstrated in female *Drosophila* that fragmented mtDNA facilitates selective clearance of mutant mtDNA through a quality control mechanism. Extracellular vesicles (EVs) have been established as crucial mediators in inflammatory processes (Hezel et al., 2017), serving as effective carriers for mitochondrial components. While larger EVs (e.g., microvesicles) can transport entire mitochondria, smaller EVs (e.g., exosomes) primarily deliver mtDNA and other nucleic acids to recipient cells (Berridge and Neuzil, 2017; Sansone et al., 2017). However, the precise mechanisms governing EV-mediated mtDNA uptake by target cells remain elusive. Microvesicles, ubiquitous in various bodily fluids, play pivotal roles in intercellular communication. Upon detachment from parent cells, they facilitate long-distance molecular transport (Turola et al., 2012). Notably, mitochondria-containing microvesicles can function as danger signal transducers. Studies have confirmed that mesenchymal stem cells and astrocytes secrete large microvesicles containing functional mitochondria, which can be recognized by epithelial cells, immune cells, and neurons (Hayakawa et al., 2016; Sinha et al., 2016). P. Sansone et al. further demonstrated that EVs can mediate complete mtDNA transfer between cells, potentially altering the endogenous mitochondrial repertoire of recipient cells (Sansone et al., 2017). Exosome biogenesis and secretion are cell type- and purpose-dependent. For instance, maximal exosome release coincides with apoptotic processes. Microvesicles express surface receptors enabling target cell recognition and subsequent membrane retention, endocytosis, or lysosomal degradation (van Niel et al., 2018). Platelets represent the most abundant and complex extracellular mitochondrial carriers in circulation. These anucleate, discoid structures-derived from bone marrow megakaryocytes-typically harbor approximately 4 mitochondria each. With nearly one trillion platelets present in human blood, their production, activation, and clearance are intimately linked to mitochondrial dynamics and interactions with other platelet organelles. These findings collectively establish that cf-mtDNA release occurs through multiple regulated pathways, with vesicle-mediated transport representing a sophisticated intercellular communication system that influences both physiological homeostasis and pathological processes, while simultaneously presenting novel opportunities for biomarker development and therapeutic.

## 3 Origins and biological significance of cf-mtDNA

During cell death, mtDNA can be actively released into the cytoplasm through selective mitochondrial membrane permeabilization. Concurrently, studies have demonstrated (Land, 2012) that mtDNA may also undergo passive release into the extracellular environment during cell death. As reported (Patrushev et al., 2004), oxidative stress-induced mitochondrial damage leads to mtDNA fragment escape into the cytoplasm through opening of the mitochondrial permeability transition pore (mPTP)-a process that can be pharmacologically inhibited by cyclosporine A. When released into extracellular spaces, these mtDNA fragments function as damage-associated molecular patterns (DAMPs). Notably, human lymphocytes have been shown to actively secrete mtDNA into extracellular compartments (Ingelsson et al., 2018), subsequently triggering inflammatory responses. This observation confirms the existence of specific regulatory mechanisms governing mitochondrial genome release. White and Kile (2015) demonstrated that viral infections can induce mtDNA stress, leading to cytoplasmic mtDNA release through pathways distinct from apoptotic mtDNA extrusion. However, the precise regulatory mechanisms controlling mtDNA translocation from cytoplasm to extracellular space remain unclear (Nakahira et al., 2015). Current evidence identifies cellular stress as the primary driver of mtDNA release. Caielli et al. (2016) proposed an alternative release mechanism requiring fusion between mitochondrial and plasma membranes, though this hypothesis awaits experimental validation. Below we systematically review potential cellular origins of mtDNA as documented in current literature.

### 3.1 Mitophagy

Mitochondria are autonomous and highly dynamic double-membrane organelles in eukaryotic cells that participate in numerous cellular processes. Cell proliferation, apoptosis, and intracellular calcium homeostasis represent several key examples where mitochondria play prominent roles (De Stefani et al., 2016; Diebold and Chandel, 2016; Giorgi et al., 2012). When external hemodynamic stresses cause mitochondrial damage, the organelles undergo degradation via the autophagy-lysosome system (Nakai et al., 2007). Autophagy involves the sequestration of cytoplasmic components into double-membrane vesicles that fuse with lysosomes to facilitate content degradation. This evolutionarily conserved process primarily serves quality control and renewal functions for cytoplasmic organelles in mammalian cells while preserving nuclear integrity. Although commonly associated with cell death, autophagy performs multiple other critical functions: i, providing nutrients during starvation (May et al., 2008); ii, serving as an adaptive mechanism to protect the heart against hemodynamic stress (Nakai et al., 2007), and iii, selectively eliminating damaged mitochondria under specific conditions (Takano-Ohmuro et al., 2000). Notably, autophagy demonstrates significant correlation with dissipation of mitochondrial membrane potential (ΔΨm) (Elmore et al., 2001), enabling selective targeting of dysfunctional mitochondria to maintain cellular energy efficiency. During mitophagic degradation of impaired mitochondria, mtDNA may be released into plasma (Oka et al., 2012; Rello-Varona et al., 2012). In summary, mitophagy serves as a crucial quality control mechanism that not only eliminates damaged mitochondria but may also contribute to the pool of circulating cf-mtDNA with potential pathophysiological implications.

### 3.2 Aging and sterile inflammation

Aging represents a complex multifactorial process, with the “mitochondrial theory of aging” proposing central involvement of mitochondria in senescence (Miquel et al., 1980). Activation of individual mitochondrial quality control pathways depends on the extent of mitochondrial damage. Aging disrupts the mitochondrial quality control axis, thereby driving senescence.

While inflammation has long been recognized as a defensive response against microbial pathogens, it is now established that chronic inflammatory responses can occur in the absence of infection-a condition termed “sterile inflammation” (Chen and Nunez, 2010). This concept describes the chronic systemic inflammatory state characteristic of aging (Franceschi et al., 2000). This paradigm shift highlights how mitochondrial-derived signals can initiate inflammation independently of pathogenic challenge.

Mitochondria play pivotal roles in sterile inflammation, which arises from redox imbalance. Under moderate inflammation, overwhelmed cellular repair systems may trigger intrinsic apoptotic cascades (Fiers et al., 1999). During severe inflammation, mitochondrial dysfunction and ROS-induced damage can instead promote necrosis, leading to release of cellular contents including intact/fragmented mitochondria and mtDNA (Fiers et al., 1999). Cell damage and death release cf-mtDNA, which functions as a DAMPs to induce caspase-1 activation and proinflammatory cytokine release (Dayama et al., 2014). Studies report that during acute trauma, damaged cells release substantial mtDNA into circulation (Zhang et al., 2010a; Zhang L. et al., 2016); this extracellular mtDNA binds Toll-like receptors (TLRs), activating NF-κB and MAPK pathways to stimulate massive release of inflammatory factors (TNF-α, IL-1, IL-6), ultimately causing acute lung injury. Additional research demonstrates cf-mtDNA can induce myocardial inflammation, leading to myocarditis and cardiomyopathy (Oka et al., 2012). Together, these findings position mitochondria-derived cf-mtDNA as a key mediator connecting cellular stress responses to systemic inflammatory conditions through multiple well-defined molecular pathways.

### 3.3 Oxidative stress

Mitochondria represent the primary source of ROS, which are generated at various organelle sites as byproducts of substrate oxidation and oxidative phosphorylation. Oxidative stress is implicated in multiple pathological processes, including cardiovascular diseases, cancer, neurological disorders, diabetes, arthritis, aging, and sepsis. Oxidative stress-mediated mtDNA damage triggers a vicious cycle of ROS production and further mitochondrial impairment, ultimately leading to apoptosis or cell death-a phenomenon termed mitochondrial catastrophe or toxic oxidative stress. Critically, oxidative stress in mitochondria promotes the oxidized release of both cf-nDNA and cf-mtDNA into circulation. In summary, mitochondrial oxidative stress serves as a key mechanistic link between cellular metabolic dysfunction and the generation of oxidation-modified circulating nucleic acids that may propagate systemic damage.

### 3.4 Exercise

Repeated exercise bouts serve as potent drivers of physiological adaptation. Training-induced adaptations are reflected through changes in contractile proteins, mitochondrial function, metabolic regulation, intracellular signaling, and transcriptional responses (Egan and Zierath, 2013). Chronic endurance training elicits diverse metabolic and morphological alterations, including mitochondrial biogenesis and muscle fiber type transitions (Bogdanis, 2012; Coffey and Hawley, 2007). Shockett et al. demonstrated that prolonged moderate-intensity exercise (90-min treadmill running at 60% VO_2_max) significantly reduced circulating cf-mtDNA levels in healthy, moderately-trained young males (Shockett et al., 2016). These findings collectively suggest that exercise modulates cf-mtDNA dynamics, potentially reflecting improved mitochondrial efficiency and reduced cellular stress in trained individuals.

### 3.5 Mitochondrial dysfunction

Emerging evidence demonstrates that mitochondrial dysfunction significantly alters cf-mtDNA release patterns and cellular communication. Dantham et al. (2016) evealed that mutations in mitochondrial protein-coding genes reduce the cf-mtDNA to cf-nDNA ratio, and that nutritional intervention with ω-3 fatty acids helps restore this balance. Studies have shown (Kansaku et al., 2018) that carbonyl cyanide m-chlorophenyl hydrazone (CCCP)-induced mitochondrial dysfunction increases cf-mtDNA secretion from cumulus-oocyte complexes (COCs). When denuded oocytes were cultured separately, CCCP treatment resulted in minimal cf-mtDNA detection in the culture medium, indicating that nearly all secreted cf-mtDNA originates from surrounding granulosa cells. Importantly, mitochondrial dysfunction in granulosa cells leads to increased cf-mtDNA secretion into the culture environment. These findings collectively highlight that mitochondrial functional status directly governs cf-mtDNA release dynamics, with important implications for both diagnostic applications and therapeutic interventions targeting mitochondrial health.

### 3.6 Cancer

The unique molecular characteristics of mtDNA offer distinct advantages over nDNA for liquid biopsy applications in oncology. Compared to the nuclear genome, the shorter length and higher abundance of mtDNA molecules significantly enhance the sensitivity and accuracy of using mtDNA alterations as molecular markers to detect rare tumor cells in body fluids. The identification of cf-mtDNA in plasma or serum samples from cancer patients has recently attracted considerable attention, stimulating research interest in its diagnostic potential across multiple cancer types. Ellinger et al. demonstrated that serum cf-mtDNA levels were significantly elevated in patients with urological malignancies (including bladder cancer, prostate cancer, renal cell carcinoma, and testicular cancer) compared to healthy volunteers, showing both sensitivity and specificity for cancer detection (Ellinger et al., 2009; Elling et al., 2012). Similarly, patients with epithelial ovarian cancer exhibited substantially higher serum cf-mtDNA levels compared to those with benign ovarian conditions and healthy individuals (Zachariah et al., 2008). These collective findings strongly suggest that tumor cells actively secrete cf-mtDNA, positioning it as a promising biomarker for non-invasive cancer detection and monitoring across diverse malignancies.

## 4 cf-mtDNA as a danger-associated molecular pattern in disease pathogenesis

The evolutionary origins and structural properties of mtDNA underpin its critical role in sterile inflammation and disease pathogenesis. cf-mtDNA has garnered significant attention as a DAMPs in cancer, trauma, and other pathological conditions. Evolutionarily derived from bacterial DNA, mtDNA shares structural similarities including double-membrane association, circular genome architecture, autonomous replication, and unmethylated CpG motifs. These conserved features enable extracellular mtDNA to mimic pathogen-associated molecular patterns (PAMPs) under cellular stress or injury conditions, functioning as potent DAMPs that are recognized by pattern recognition receptors (PRRs) of the immune system to activate innate immunity and inflammatory responses.

Notably, PAMPs and DAMPs are frequently detected by overlapping receptor systems, explaining the mechanistic parallels between sterile inflammation and pathogen-induced inflammation. Collins et al. (Collins et al., 2004) first demonstrated the immunogenicity of mtDNA in 2004, showing that murine splenocyte exposure to mtDNA triggered tumor necrosis factor (TNF) secretion, while joint injection induced arthritis. Subsequent studies (Zhang et al., 2010b; Gan et al., 2015; Tsuji et al., 2016) have consistently confirmed that exogenous mtDNA administration elicits both localized and systemic inflammatory responses. Specifically, mtDNA DAMPs stimulate neutrophils to activate immune cells, promoting proinflammatory cytokine release and propagating injury to distant organs (Simmons et al., 2013). The inflammatory cascades are primarily mediated through three key pathways: i, TLR9 recognizes hypomethylated CpG motifs in mtDNA, engaging MYD88 to activate MAPK and NF-κB pathways (enhanced by IRF7) (Itagaki et al., 2015); ii, NLRP3 inflammasome assembly through mtDNA binding; and iii, cGAS-STING-IRF3 signaling axis activation that amplifies interferon-stimulated gene (ISG) expression. Our preliminary data corroborate that cf-mtDNA engages cell surface TLR9 to activate NF-κB/MAPK pathways, driving both inflammation and apoptosis (Liu et al., 2020). Furthermore, mtDNA directly interacts with activated leukocytes to modulate antimicrobial responses (Zhang et al., 2010a). While mtDNA DAMPs clearly function in intercellular signaling, their generation and release mechanisms remain enigmatic. Emerging evidence (Kuck et al., 2015) reveals an association between mtDNA and mitochondrial TFAM, with TFAM acting as a TLR9 co-factor to amplify TNF-α release (Julian et al., 2013). In conclusion, cf-mtDNA represents a unique evolutionary relic that bridges mitochondrial dysfunction to systemic inflammation through multiple parallel immune activation pathways, offering novel therapeutic targets for DAMPs-mediated pathologies.

## 5 cf-mtDNA as a clinical biomarker: biological rationale and methodological advances biological feasibility and clinical relevance of cf-mtDNA

cf-mtDNA has gained increasing attention as a clinically informative biomarker due to its unique biological properties, broad distribution in body fluids, and close association with mitochondrial dysfunction, inflammation, and cellular stress. Beyond plasma, cf-mtDNA has been detected in pleural effusions (Sriram et al., 2012), cerebrospinal fluid (CSF) (Liimatainen et al., 2013), synovial fluid (Leon et al., 1981), and other extracellular compartments, underscoring its versatility for minimally invasive disease monitoring. Compared with nDNA, mtDNA displays enhanced resistance to nuclease-mediated degradation (Manuelidis, 2011), enabling its stable persistence in extracellular environments and facilitating reliable detection.

Accumulating clinical evidence demonstrates disease-specific alterations in cf-mtDNA abundance and structure. In neurological disorders, CSF cf-mtDNA levels are markedly reduced in Alzheimer’s disease (Podlesni et al., 2013), Parkinson’s disease (Pyle et al., 2015), and advanced multiple sclerosis (Lowes et al., 2019), with the degree of reduction correlating with neuronal dysfunction and disease progression (Gambardella et al., 2019). In cardiovascular disease, elevated plasma cf-mtDNA levels have been consistently observed in coronary artery disease (Liu et al., 2016) and acute myocardial infarction (Bliksoen et al., 2012), reflecting tissue injury and inflammatory activation. In oncology, cf-mtDNA alterations show diagnostic and prognostic relevance across multiple malignancies, including ovarian cancer (Meng et al., 2019), head and neck cancer (Jiang et al., 2005), and lung cancer (Hosgood et al., 2010). Additional associations have been reported in trauma (Lam et al., 2004), infection and inflammation (Arshad et al., 2018), toxic or oncogenic exposures (Budnik et al., 2013), and aging (Pinti et al., 2014), positioning cf-mtDNA as a dynamic, multi-system biomarker.

Notably, cf-mtDNA signatures may vary by disease context. In breast cancer, conflicting reports describe both increased (Mahmoud et al., 2015) and decreased cf-mtDNA levels, likely reflecting differences in disease stage, analytical methods, and preanalytical handling. Recent evidence indicates that cf-mtDNA is enriched within EVs, particularly exosomes, compared with freely circulating DNA (85), highlighting vesicle-mediated mtDNA transport as a biologically relevant component of cf-mtDNA signaling. However, the inability to reliably distinguish free from vesicle-encapsulated mtDNA during isolation remains a major source of variability, emphasizing the need for standardized workflows.

A central technical limitation in cf-mtDNA analysis is interference from nuclear mitochondrial pseudogenes (NUMTs). Extensive integration of mtDNA-derived sequences into the nuclear genome—up to 612 loci with high sequence homology—has been documented (Chiu et al., 2001), with some NUMTs approaching full-length mtDNA (50). Without NUMTs-aware assay design, PCR-and sequencing-based approaches may generate false-positive signals. Bioinformatic identification of mtDNA regions with minimal nuclear homology has enabled the development of mtDNA-specific primers (e.g., hmito3, hmito5) alongside nuclear controls (hB2M1, hB2M2) (Malik et al., 2011). Complementary strategies, including Φ29 polymerase–mediated rolling circle amplification, targeted mtDNA sequencing, and pre-PCR template dilution, further mitigate NUMTs contamination (Calvignac et al., 2011; Li et al., 2012; Wolff, 2014). Importantly, the reported mtDNA-specific primers (e.g., hmito3, hmito5, ND1/ND2-based assays) were designed with short amplicon lengths (typically <100 bp), specifically accommodating the highly fragmented nature of cf-mtDNA. This design strategy minimizes NUMTs interference while preserving efficient amplification of short cf-mtDNA fragments, thereby avoiding systematic underestimation of mitochondrial targets in cell-free samples.

Preanalytical variability represents an additional critical determinant of cf-mtDNA measurement accuracy. Blood processing protocols substantially influence cf-mtDNA quantification (Chiu et al., 2003; Ng et al., 2002). Single-step centrifugation yields artificially elevated mtDNA levels due to residual cellular debris, whereas double centrifugation more effectively produces cell-free plasma (Baysa et al., 2019). Moreover, cf-mtDNA exists in both free and vesicle-associated forms, with filtration or ultracentrifugation markedly reducing detectable mtDNA levels (Wolff, 2014). These findings underscore the necessity of standardized centrifugation and vesicle-handling procedures. Structural differences between mitochondrial and nuclear genomes further introduce “dilution bias” in mtDNA/nDNA ratio-based quantification (Malik et al., 2009). Template fragmentation by ultrasonication effectively eliminates this bias, enabling consistent and accurate mtDNA copy number assessment across dilution conditions (Chiu et al., 2001).

Collectively, cf-mtDNA is supported by strong biological plausibility and growing clinical evidence, but its translation into routine diagnostics depends on rigorous standardization of preanalytical, analytical, and computational methodologies.

## 6 Advances in cf-mtDNA extraction and high-throughput sequencing

### 6.1 cf-mtDNA extraction: Optimization and standardization

Reliable cf-mtDNA analysis requires optimized extraction strategies that preserve short, low-abundance fragments while minimizing technical bias. Extraction methods have evolved from phenol–chloroform protocols to silica column–based kits and, more recently, automated magnetic bead platforms. Early organic extraction methods were labor-intensive and prone to loss of short cf-mtDNA fragments (Zhang R. et al., 2016). Column-based kits, such as the QIAamp Circulating Nucleic Acid Kit, improved short-fragment enrichment and revealed elevated cf-mtDNA levels in head and neck squamous cell carcinoma (Kumar et al., 2017), although recovery of fragments <100 bp remained limited.

Magnetic bead–based methods now dominate due to scalability and automation compatibility. Protocol optimization has increased cf-mtDNA recovery by up to 95-fold while reducing edge effects in high-throughput platforms (Ware et al., 2020). Sample-specific adaptations are essential: CSF samples, characterized by extremely low cf-mtDNA abundance, require high-sensitivity kits with spike-in controls (Takousis et al., 2022), whereas urinary cf-mtDNA—largely vesicle-associated—necessitates pre-clearing steps such as centrifugation or filtration (Kim et al., 2019).

Integration of extraction and detection has further reduced sample loss. The MitoQuicLy approach enables direct plasma lysis and cf-mtDNA quantification without prior extraction, producing results comparable to conventional methods with reduced cost and processing time (Michelson et al., 2023). Equally important, plasma preparation protocols that minimize platelet activation significantly reduce artifactual mtDNA release; platelet-stabilized processing decreased cf-mtDNA levels by up to 67-fold in healthy individuals (Roch et al., 2024).

Standardization of extraction workflows remains essential for clinical translation. EDTA-based blood collection and prompt double centrifugation are recommended (Arshad et al., 2018), with low-temperature, low-activation conditions to limit platelet-derived mtDNA contamination (Roch et al., 2024). Magnetic bead systems using carboxyl-modified beads and optimized salt conditions enhance recovery of ultra-short fragments by up to 11.5-fold (Zhang R. et al., 2016), while automated 96-well platforms reduce inter-assay variability to <5% (Kumar et al., 2017). Importantly, kit performance is sample dependent: the Maxwell RSC ccfDNA Plasma Kit outperformed QiAamp MinElute kits in endometriosis plasma (Huebner et al., 2021), whereas Norgen kits showed superior spike-in recovery in CSF(95).

### 6.2 High-throughput sequencing and analytical advances

Digital PCR (ddPCR) has emerged as the reference method for cf-mtDNA quantification due to its absolute measurement capability and resistance to PCR inhibition, with detection limits approaching 1 copy/μL when targeting ND1 (Ye et al., 2017). Primer design targeting conserved regions (e.g., ND1, ND2) minimizes false negatives due to deletions or polymorphisms (Ogarkov et al., 2022). Multitarget strategies reduce batch effects and improve diagnostic performance; combined ND1 and MT-CO1 detection achieved an AUC of 0.865 in COVID-19 patients (Hepokoski et al., 2022). Harmonized magnetic bead extraction combined with ddPCR reduced inter-laboratory variability from ∼30% to <5% in multicenter studies (Harrington et al., 2019).

Next-generation sequencing (NGS) enables comprehensive cf-mtDNA profiling, including mutation spectra, fragmentation patterns, methylation status, and tissue origin. Exosomal cf-mtDNA spans the full mitochondrial genome and exhibits longer fragment lengths than free plasma cf-mtDNA in hepatocellular carcinoma (Li et al., 2020). In renal and colorectal cancers, enhanced fragmentation of mutant cf-mtDNA improves mutation detection following size selection (Zhou et al., 2022). Methylation-based methods, such as cfMeDIP-seq, further expand the cf-mtDNA biomarker landscape (Shen et al., 2019). NGS also facilitates tracking of cf-mtDNA origin, as demonstrated by donor-derived mtDNA SNPs in bone marrow transplantation recipients (Malik et al., 2009).

NGS is particularly powerful for detecting low-level heteroplasmy. Plasma cf-mtDNA mutation profiles reflect intratumoral heterogeneity in head and neck cancer (Kumar et al., 2017), while exosomal cf-mtDNA enables detection of variants below 1% allele frequency (Bjornetro et al., 2023). Integration of fragmentomic features, including end-motif patterns, substantially enhances diagnostic accuracy, with reported AUC values up to 0.983 in hepatocellular carcinoma (Liu et al., 2025).

Library preparation must accommodate short, degraded, and low-input cf-mtDNA. Efficient protein removal, inhibitor clearance, and pre-concentration are essential, particularly for CSF samples (Zhang R. et al., 2016; Kumar et al., 2017; Takousis et al., 2022). Fragmentation strategies are tailored to initial fragment size, with transposase-mediated tagmentation offering rapid library construction while preserving fragment uniformity (Shen et al., 2019; Bruinsma et al., 2018). Adapter ligation efficiency is improved using high-fidelity ligases and single-stranded adapters for ultra-short fragments (Raine et al., 2017). Low-cycle PCR amplification with high-sensitivity polymerases preserves library complexity (Bruinsma et al., 2018; Parkinson et al., 2012). Rigorous quality control—including fragment size profiling, library quantification, and NUMTs contamination assessment—is indispensable (Zhou et al., 2022; Liu et al., 2025).

**FIGURE 1:**
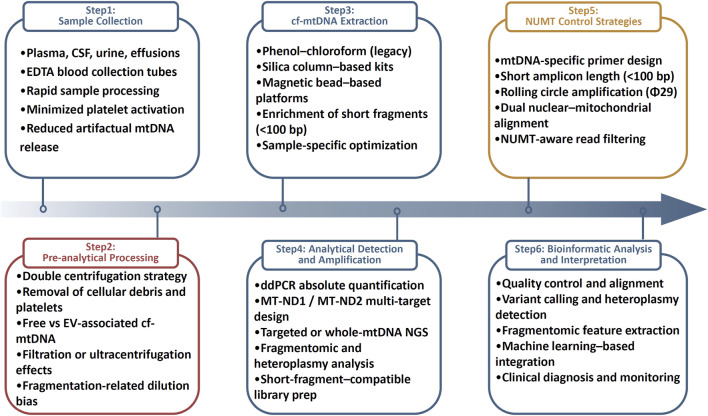
Standardized pre-analytical and analytical workflow for cf-mtDNA analysis. cf-mtDNA analysis involves sequential pre-analytical and analytical steps that shape data quality and comparability. The workflow encompasses biofluid collection with platelet-stabilized handling, pre-analytical processing including centrifugation, vesicle management, and control of fragmentation-related bias, and optimized extraction of ultra-short fragments. Downstream detection relies on ddPCR and next-generation sequencing, supported by short-fragment–compatible library preparation. Experimental and computational strategies are applied to mitigate interference from NUMTs, enabling robust bioinformatic analysis and clinical interpretation of cf-mtDNA.

Bioinformatic analysis requires specialized pipelines to address short read length, high copy number, and NUMTs interference. Key steps include stringent quality filtering, optimized mitochondrial alignment, parallel nuclear genome mapping for NUMTs removal, and advanced variant calling for heteroplasmy detection (Zhang R. et al., 2016; Zhou et al., 2022). Fragmentomic and machine learning–based integration of multidimensional features further improves diagnostic robustness and cross-study comparability (Zhang et al., 2021). Ongoing advances in library chemistry, targeted mtDNA capture, PCR-free long-read sequencing, and standardized computational frameworks continue to reduce analytical bias (Harrington et al., 2019; Zhou et al., 2022; Raine et al., 2017). An overview of the cf-mtDNA analytical pipeline, from biospecimen collection to downstream molecular analysis, is shown in Figure 1.

## 7 Conclusion

In summary, circulating double-stranded mtDNA molecules present in bodily fluids have demonstrated remarkable diagnostic and prognostic value since their discovery. Current evidence indicates these molecules originate primarily through cellular oxidative stress, mitochondrial dysfunction, and autophagic secretion. However, widespread clinical adoption necessitates rigorous standardization of preanalytical variables-particularly sample handling and cf-DNA extraction methods to ensure reproducibility across laboratories and enable meaningful interstudy comparisons.
